# A tool for Swarm satellite data analysis and anomaly detection

**DOI:** 10.1371/journal.pone.0212098

**Published:** 2019-04-29

**Authors:** Vyron Christodoulou, Yaxin Bi, George Wilkie

**Affiliations:** School of Computing, Faculty of Computing, Engineering and the Built Environment, Ulster University, Newtownabbey, United Kingdom; Southwest University, CHINA

## Abstract

In this work we introduce a system pipeline for the analysis of earth’s electromagnetic field that is used to analyse precursors to earthquakes. Data gathered by the Swarm satellites are used to present the utility of our system. Our objective is to provide a streamlined method to analyze electromagnetic data over a region and investigate the relationship of precursory signals to seismic events. The process follows three distinct stages: data extraction, data pre-processing and anomaly detection. The first stage consists of the region selection and data extraction. The second stage consists of four different pre-processing methods that address the data sparsity problem and the cause of artificial anomalies. The last stage is the Anomaly Detection (AD) of the Swarm satellite data, over the investigated region. The different methods that are implemented are known to perform well in the field of AD. Following the presentation of our system, a case study is described where the seismic event of 6.2 M_w_ is in Ludian, China and occurred on 3^rd^ August 2014. The event is used to present the usefulness of our approach and pinpoint some critical problems regarding satellite data that were identified.

## 1 Introduction

Visualization of time series data focuses on the understanding of patterns, anomalies and variations in the context of data mining. For a long time the only available method for time series analysis was the visualization and subsequent analysis from a human expert [1], [2]. The expert becomes accustomed to the visualisation process and is able to understand patterns and distinguish between normality and abnormality in time series data.

Since the advent of the information era, the visual inspection of such time series by human experts is still a useful approach for verification or data labelling purposes. However, the continuous growth of data availability makes the manual analysis task slow and impractical. The automation of this process contributes to the quick, reliable and efficient detection of anomalous patterns in time series. Moreover, the automation advances the exploration and deeper understanding of the causes of unexpected variations. In combination with the knowledge of human experts, a system can outweigh the benefits of only a human visual approach. In geoscience, as in other fields, data growth will be especially crucial to understand the causes of such variations with the aim of making possible predictions in the future.

Satellites sensors have been used to provide a more reliable source of electromagnetic (EM) data and give a bigger picture of analysis of time series models for many years. Swarm satellites’ sensors complemente the magnetic field variations with plasma, accelerometer and electric field measurements. The Swarm satellite constellation [3], monitors earth’s magnetic field (EMF) and it consists of three satellites. Satellites A and C fly side by side and at an altitude close to 450km and a distance of 100km from each other. Swarm B flies at an altitude close to 510km on an orbit almost perpendicular to that of A and C. It was thought that in order to address the problem of data availability a data combination from the satellites that fly in parallel could be used. The data density alone is an opportunity to address some of the limitations present in the literature and focus on algorithm parameter selection, Anomaly Detection in a short time window and simplicity as described in [4]. Other limitations such as data quality, data gathering frequency and data integrity have also been a problem described in [5] that do not allow reliable forecasting. It is our objective to address those problems and propose new solutions.

The evaluation and analysis of Swarm data is the focal point of our study. A visual analysis of time series data is nowadays an inefficient task that is better handled by the addition of automated methods [6]. Therefore, we propose an automated analysis tool that streamlines the process of Swarm data by presenting different methods to handle data sparsity and other data quality limitations. AD methods are here used to identify possible precursory variations in the earth’s magnetic EMF due to seismic events.

Aside from the use of visual feedback or simple statistical analysis, the main question is the accuracy of AD and to find a reliable way to eliminate any possible interference from other sources (human, space or other natural causes). Moreover, not many software packages exist that streamline the process from satellite sources and perform data analysis towards AD. The adoption of such software packages is slow because there is no standard format that exists in either satellites or terrestrial sources. This fact makes data extraction a painstaking process. Furthermore, frequently there is missing data, data quality issues, and general data availability problems that pose a significant obstacle to the analysis and streamlining of a process. Most existing solutions circumvent those problems by focusing on each stage one by one. It is then understandable, that this makes the processing slower and does not show the whole picture of where the difficulties in processing lie. It is our objective to do so and provide an insight into how to address some of the previously mentioned problems. The proposed prototype that combines data extraction, visualization and AD, adds another integral part to the body of work concerning satellite data sources and EM variations for studying seismic events.

The paper is organized as follows. In Section 2, the related work is discussed and how this work fits in the visualization analysis scope. Section 3 describes the architectural design of the prototype and the functional design. Section 4 discusses the nature of the real data used. Section 5 presents a real world application and its results are discussed. In Section 6 we give a summary and further possible directions regarding the future work and possible solutions within the field of data mining. Finally, a Appendix section is included at the end that defines the main terms used in our work.

## 2 Literature review

The movement of earth’s lithosphere releases large amounts of energy through seismic events. Even though those large seismic events are rare, they provide a unique opportunity to test the hypothesis that seismic events could be anticipated after EM variations. Nowadays, the large breadth of sensors available provide the necessary data that make this investigation easier than ever before. There is a large body of scientific evidence that implies the existence of precursory signals in different datasets. Most studies are predicated on the fact that EM anomalies can occur prior to seismic events [7][8]. Moreover, they confirm the presence of EM disturbances prior to seismic events within an effective distance (ED) of up to 4000km where an anomaly can be detected [9].

Different kinds of data have been used to investigate this relationship. In an early study, Lie et al. uses the Total Electron Content (TEC) measurements using the interquartile (IQR) and median range as thresholds to detect any precursory anomalies in the ionosphere [10]. TEC data were also analyzed with an Artificial Bee Colony Optimization algorithm in [4]. More recently in [11] the authors propose a statistical approach, the Geometric Moving Average for change detection in Outgoing Longwave Radiation with promising results. Variations in the very low and ultra low frequency bands have been the focus in [12], [13].

Satellites have provided bigger and more accurate datasets to assess the connections of precursors and seismic events. Data from the CHAMP and DEMETER satellites were used to study ionospheric perturbations in [14]. In [15], the ionospheric ion density by the DEMETER satellite was investigated. Ultra Low Frequency (ULF) signals in that range have been explored as potential precursors to seismic events since several decades ago [16]. More recently, adding to that theory, similar studies with Swarm data have published positive results regarding EM variations as precursors to seismic events. [17, 18] with the focus being the Pc3(22-100 mHz) wave range [19].

The constant data growth has caused need for more elaborate methods for the analysis of time series data, in the name of reducing and approximately modelling the original time series. A common family of time series representation methods are approaches that map numbers to symbols directly from the time series without any transformation to another domain. Most methods used in our prototype use some form of symbolic approximation. The following methods are implemented as part of our system: HOT-SAX [20], 1D-SAX [21], Fuzzy Shape-based [22] and CUSUM-EWMA (CE) [23]. The inclusion of such methods into software models has not been a priority. However, a few software solutions exist that provide both a visualization tool and an AD process and are going to be discussed below.

As far as streamlining the process of data processing and data AD a few tools have been developed but do not go so far as to combine the two. A visualization tool for Swarm data is supplied by the European Space Agency (ESA) [24]. The amount of data gathered everyday from such missions provides a first class opportunity to analyze and visualize geomagnetic data in an open source environment. The user can select which sensor’s data and on which dates to visualize. There is also the ability to generate histograms and a time series view of the data and analyse them manually.

Elsewhere, an AD tool for pattern discovery in time series was implemented in [25]. GrammarViz is a tool that presents a visualization and a grammar rule mining of time series data. It utilizes the SAX approach for symbolic representation and discretization and it is able to find subsequences of variable length. It is also useful to classify patterns based on their symbolic representation. However this tool addresses only the AD requirement in time series analysis.

The project COPEPOD [26] is another time series analysis tool. It analyses phytoplankton data gathered by satellites which are processed into time series graphs. It is used for long-term ecosystem monitoring. This tool fulfils only the visualization requirement in data analysis. It is useful to see that many of these approaches would benefit from a unified framework that would provide forecasting, AD or any other analysis method.

A recent work in the field of EM variations acting as precursors to earthquakes has been carried out by [27] based on a theoretical framework proposed in [28]. This work lays the foundations of data analysis in a geophysical environment on the earthquake-ionosphere coupling. They present promising results but their analysis is only based on ground-based stations. The main difference in our work is that gathering data from satellites introduces more problems as the data is not continuous in the region we investigate.

TIMESAT, a tool for processing satellite time series sensor data based on their seasonality was proposed in [29]. The use of MODIS sensor data has generated a number of applications for both visualization [30] and AD. The premise of this visualization method is that the user is required to create a grid of interest for data extraction, something similar to our prototype. Using MODIS data, a method called Breaks For Additive Season and Trend (BFAST) was developed to monitor changes in land use and performs change detection in [31]. Carrying the work forward, a toolbox for downloading and processing MODIS data has been developed in [32].

Taking the above into account it becomes apparent that the research community focuses on (a) either the data extraction and visualization from different data products or (b) in the data analysis. This happens in two distinct stages without a streamlined and unified framework. Therefore, the two stages of data analysis are considered as two entirely different processes. This is due to the different formats available that do not allow the existence of a global framework that can handle all kinds of satellite or terrestrial data. This causes difficulties in assessing the utility of data mining methods because there are far too many issues ranging from different kinds of data, problems with data quality and data sparsity. In this work it will be shown that the creation of a prototype which bridges the gap from data extraction to algorithmic processing for AD is a pertinent issue that has not been explored in the literature.

More importantly, none of the above cases considers the triptych of data extraction, data analysis and AD into a single system. It is our interest to be able to address this problem by looking at it through a specific data tool. This way we are able to focus on all the stages of geophysical data analysis in satellite data and will be able to better pinpoint where the problems from that perspective lie in every stage of the data analysis process.

## 3 Design

### 3.1 Architectural design

**Fig 1:**
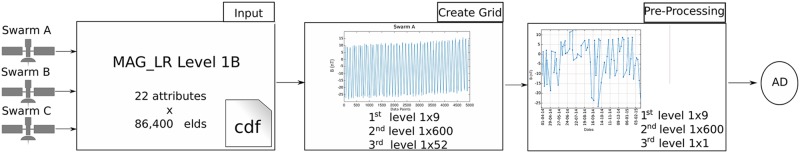
Data structure throughout the different stages of the process.

An overview of the proposed framework together with the data structures in the processing stages is shown in Fig 1. The tool is comprised of three distinct functional components: (i) data extraction, (ii) pre-processing and (iii) AD. Each output provides the input to the next stage in a streamlined process. In the following subsections each stage will be described in detail.

#### 3.1.1 Data extraction

In this work, the magnetic Vector Field Magnetometer (VFM) Level 1B(1Hz) data were used. All data were downloaded from [33] and are stored in cdf (Common Data Format), which is used for the storage of scalar and multidimensional data [34]. Each cdf file is comprised of 22 attributes, each of which has 86,400 records. That is equal to one reading per second in a single day, provided there are no data gaps. The fields of interest to our study are the date of the measurement, the longitude, the latitude and Magnetic Field Intensity (MFI) from the VFM frame. The resolution of the VFM works in a per second basis and each reading measures the intensity of EMF. In its raw format the EMF is a vector quantity. It has three orthogonal strength components (*b*_*X*_ North, *b*_*Y*_ East and *b*_*Z*_ vertical), which describe the directions and their respective strengths of intensity. In order to obtain the total intensity of the magnetic field we need to convert the three axis components to a single intensity given by Eq 1,

$$\begin{array}{c} |\vec{B}|=\sqrt{b_{X}^{2}+b_{Y}^{2}+b_{Z}^{2}} \end{array}$$ (1)

**Fig 2:**
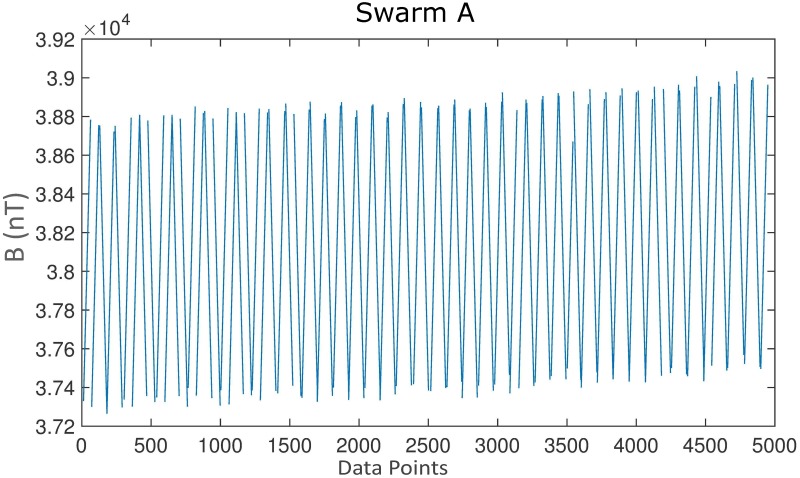
Raw data before pre-processing and magnetic field removal.

Data extraction includes the definition of the region under investigation. Each satellite overpass has close to 100km distance from the next. The region under investigation also alludes to the data gathering problems the Swarm trajectories cause. For a deeper understanding, Fig 2 shows the raw plot of data from the center-point grid without any pre-processing. The periodic oscillation is due to the latitudinal difference on the satellite orbit. When the satellite approaches the equator, i.e. the latitude is close to zero degree, the magnetic field is getting the weakest, whereas approaching the poles, i.e. the latitude is close to ±90 degree, the magnetic field is getting the strongest.

We notice two main problems in: (a) Irregular gaps in readings (the satellite might not pass from the defined Grids), or (b) Irregular number of readings per day due to the satellite’s overpass. This introduces problems such as irregular patterns in the time series. Fig 4, shows two different cases of satellite overpass. One complete for each Grid and one incomplete, that enters into neighboring Grids. To balance these problems and give equal weight to each day with readings, four different pre-processing methods are introduced in the second stage. Last but not least the processed outputs are fed to the final AD stage.

**Fig 3:**
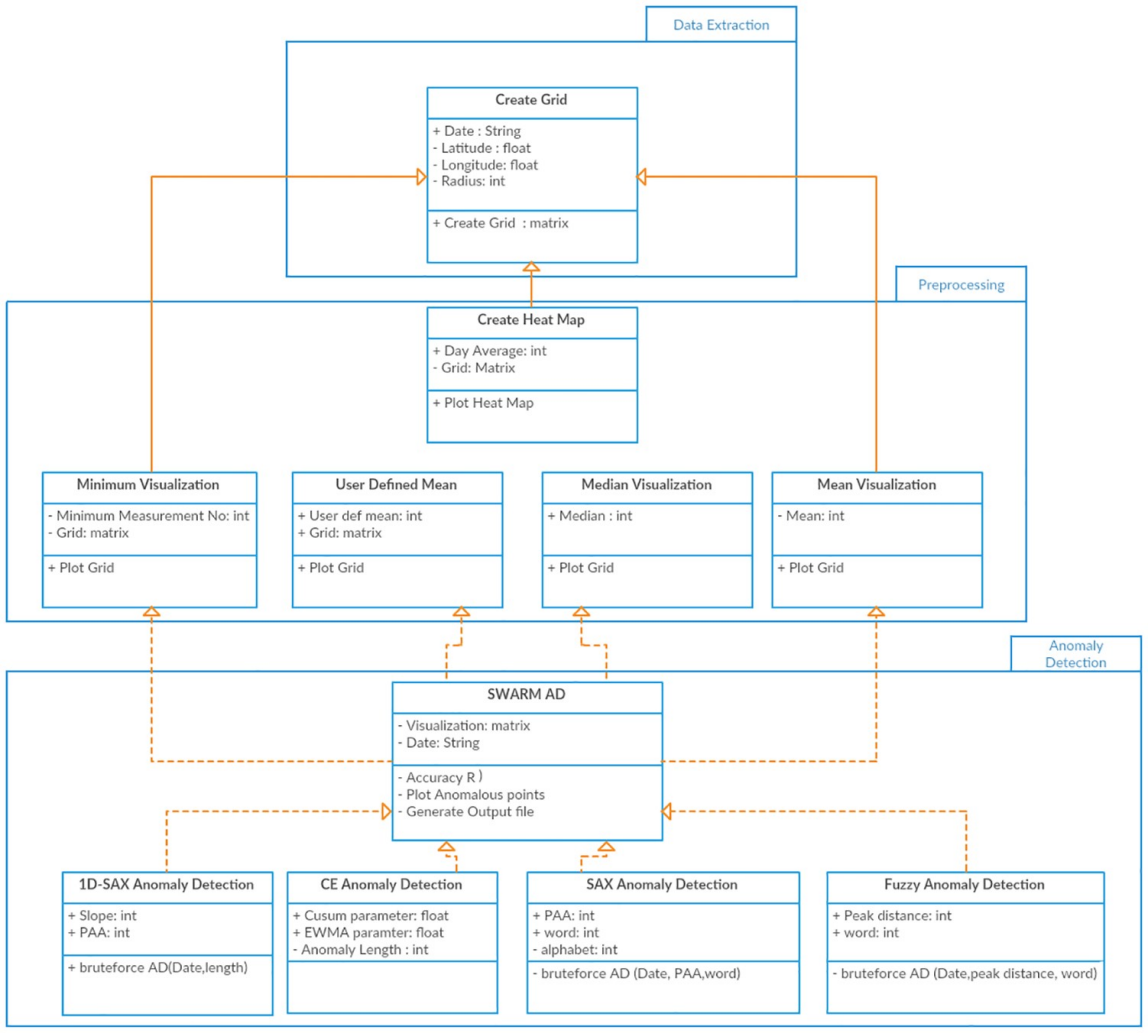
Class diagram of the proposed prototype.

**Fig 4:**
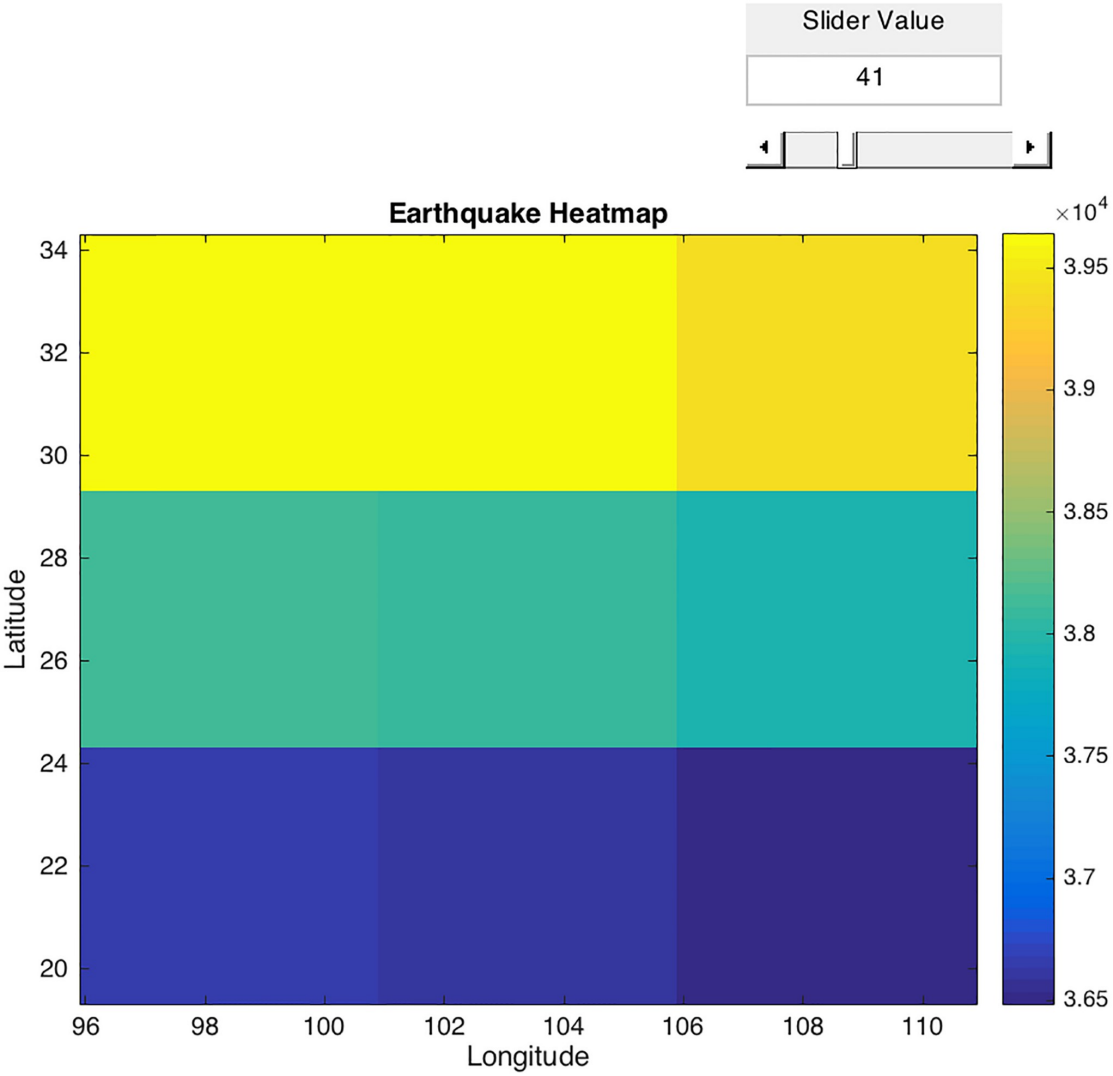
The intensity heatmap created with the default value of 5 days overlayed with the region under investigation.

The architectural design of the prototype can be seen in Fig 3, in which a class diagram of the processing stages is presented. The proposed streamlining prototype consists of three distinct stages: (a) Data Extraction and study area definition by the *Create Grid* function, (b)Data Pre-processing and (c) Anomaly Detection. The first and an integral part of the algorithm is the extraction of the time series sequence from the area in which we want to conduct our research. Every other component is based on the data extracted by the first stage. This function accepts as inputs the parameters of the seismic events. This includes its coordinates: longitude and latitude, the radius and the date when the seismic event occurred. Its function is to extract the data we require from the already imported Swarm files to the system. It then compares them against the satellites’ longitude and latitude overpass in order to place and extract each measurement to the corresponding reference Grid as shown in Fig 4.

In order to implement the square Grid, the function has to set the the limits of the Grid and extract the relevant data. The center-point of the study area is the epicenter of the seismic event. By using the coordinates as the center-point, a square grid with dimensions corresponding to the user’s set radius is created. Each degree corresponds to 100km. After creating the grid, the measurements have to be aligned in the correct order. This problem occurs due to missing dates in our dataset. Dates without data at all caused by instrumentation errors cause gaps in the time series. Moreover and more importantly, each date has a day and a night cycle. A satellite might pass from the same square Grid twice within the same date. Because there is a natural diurnal variation in the intensity of the geomagnetic field during different times, if those measurements are aggregated important information is lost during the aggregation phase. Therefore each single date has to be composed of two consecutive data points: one for daytime measurements and one for night-time.

Once the above problems are addressed the vector that includes all the measurements of the square Grid is created and is ready for analysis. Data sparsity is another problem that has to be addressed by our tool. The irregular overpasses of the satellites (when an incomplete pass occurs) over a square Grid creates a data integrity problem. The Swarm satellites have a 4 day revisit period which can be higher if the satellite makes an overpass outside the predefined square grid. For that reason, some well defined aggregation methods had to be implemented. Each one of them may or may not introduce artificial anomalies. This is clearly visible in the visualization stage, where the user is able to select which of the available data pre-processing methods they want to proceed to the AD stage.

The problems described show why the pre-processing stage is so important. The user can select which aggregation method to apply. The aggregation method does not aggregate together the dates with measurements, but it aggregates the measurements gathered within the same daytime or nighttime cycle. It is required because satellites gather measurements with different amount of data per each pass. Throughout the pre-processing stage, inconsistent measurements were causing anomalies. For example, it was noticed that if there are only 5 seconds of measurements in a nighttime cycle and 60 seconds of measurements in the next nighttime cycle an artificial anomaly is created due to the inconsistent number of readings. We circumvent artificially induced anomalies by implementing four different pre-processing methods.

#### 3.1.2 Pre-processing methods

As mentioned earlier, we expect to discover anomalies caused by seismic events in the ULF band of the signal and specifically in the range of 22-100 mHz, a type known as Pc3. Therefore, following the data extraction and the careful data munging, the next step is to bring into focus the Pc3 type waves. In this case we calculated the CHAOS geomagnetic model [35] in conjunction with applying a high pass (16 mHz) Butterworth filter, in order to filter out the main magnetic field, the ring current and to minimize the effect of any other interference/noise. The final AD is going to be performed on the residuals. Actually the problem of different strengths of intensity over different altitudes could be corrected by a global model, such as CHAOS. This is very important to the sensitivity of the algorithms because each small variation causes a significant change in the time series.

The pre-processing methods give a picture of the data quality we have in an instance. In this section, we performed different aggregation methods in order to validate the algorithms and test the possibility of introducing artificial anomalies when an aggregation method in the residuals is applied. It is shown that there is a slight variation that it is difficult to assess visually for potential anomalies. It was decided that the mean method will be used for the experimental section.

**Mean Visualization**: This method takes the mean for each date with measurements.

**Minimum Visualization**: This method can be thought as a local mean within each Grid. It first calculates what is the smallest number of readings per each Grid, that is the day with the smallest overpass. It then calculates the mean using the minimum number, that might be different for each Grid, separately.

**Median Visualization**: This function takes the median for each date with observations. Because in most cases we have 52 to 53 readings for a full overpass the median and the mean have similar results.

**User Defined Mean**: This function gives the user the ability to select how many data points they want to aggregate per each day and evaluate the time series for artificial anomalies visually. It is a form of global mean that applies to every Grid. However, if this mean is higher than the available measurements within a Grid’s date then this smaller number, that represents the smallest amount of readings available in this date, is selected instead of the user selected mean. The default value is the five first samples.

**HeatMap**: The notion of average is different when the user selects the heatmap function. The heat map function only uses one value for each grid and that is the mean. The user selects a value of how many dates the algorithm will take the mean. The usefulness of the heat map is based on the mean number the user sets. Fig 4 uses the default value of 5, meaning we aggregate sets of 5 days. It represents different EMF levels in each grid. In that case, it helps to visually detect intensity variations in the investigated region during the 35^th^ day from the start of the observations, on 5^th^ May. Due to the limitations imposed by the orbital paths of the satellites there are many dates within the grids that have no data. A small aggregation value will have many empty valued cells. Selecting a larger mean value will have less empty cells and will give a better overall picture of the difference between dates. The heatmap function helps the user understand the magnetic variations per aggregation basis.

#### 3.1.3 Anomaly detection

The output of the pre-processing method is used as the input to any of the selected AD methods. In Fig 1 the structure of the data with the mean as the preprocessing method is shown. Each dataset in its final form before AD consists of: (a) the number of grids based on the selected region, (b) the number of dates (both day and night) in separate data points that we have a reading, and (c) the final EMF per each day or night aggregated by the selected pre-processing method. All other data preprocessing methods have the same data structure with slight variations in the intensity of certain dates, as seen from the figures. A brief description of the AD methods is given in the Appendix section.

### 3.2 Functional design

#### 3.2.1 Tool interface design

**Fig 5:**
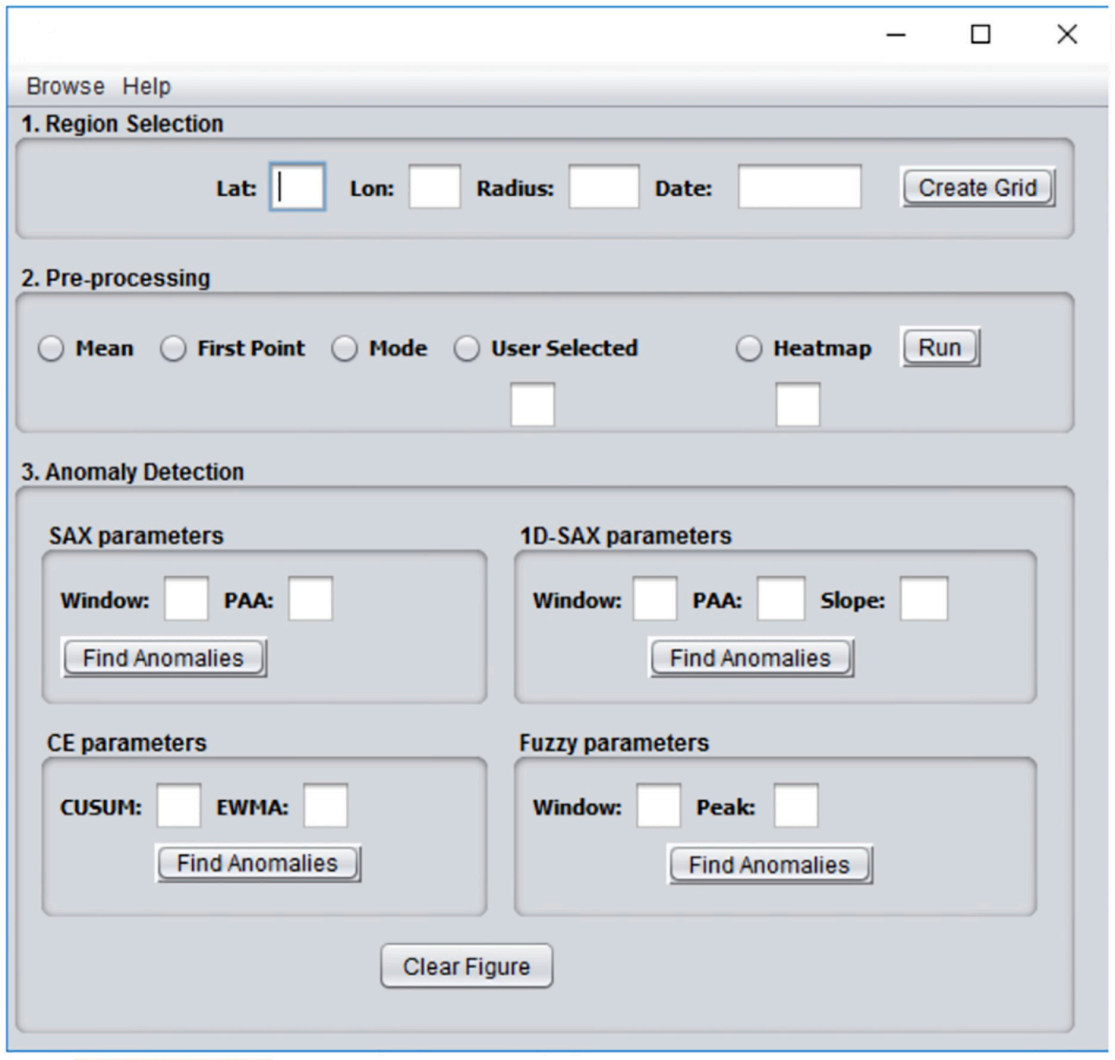
GUI of the developed software tool.

A view of the GUI is shown in Fig 5. A brief description of its parameters is given below.

**Latitude**: A float data type in the algorithm is the geographic coordinate that specifies the north-south position of the epicentre of the seismic event.

**Longitude**: A float data type in the algorithm is the geographic coordinate that specifies the east-west position of the epicentre of the seismic event.

**Date**: In DD/MM/YYYY format is the date that the seismic event under investigation occurred. We are interested in anomalies that occurred before and after the seismic event. This will allow us to understand the influence of such events to the geomagnetic field.

**Radius**: A float data type can be of value [1..*N*] with N being up to a few thousands of kilometers based on [9]. The Radius defines the distance from the epicenter to the edge of the Grid.

In Table 1 we provide the parameters’ working range for each method. This working range is based on hundreds of experiments in benchmark data [20, 22].

**Table 1:** Ranges of parameters for AD.

	CE (K,λ)	Fuzzy (P,w)	1D-SAX (p,s,w)	SAX (p,w)
Parameters	(0.1-10.0,1-N)	(1-10,1-10)	(2-8,2-8,2-8)	(1-10,1-10)

For CE the range of the λ value has a direct relation to the size of the input dataset because it denotes the amounts of historical values the algorithm is going to take into account.

For the Fuzzy Shape-based method, the peak distance has to be selected in direct proportion to the size of the input dataset. The peak distance parameter affects the selectivity and the final number of peaks that describe the reduced, after the pre-processing, signal.

For SAX, all parameters have to be selected in proportion to the size of the input dataset but there is no other requirement.

1D-SAX can accept only parameters on the power of two. The conversion stage to symbols is based on a binary conversion. The PAA has to be of a larger value than the slope.

#### 3.2.2 Time sequence of functions

**Fig 6:**
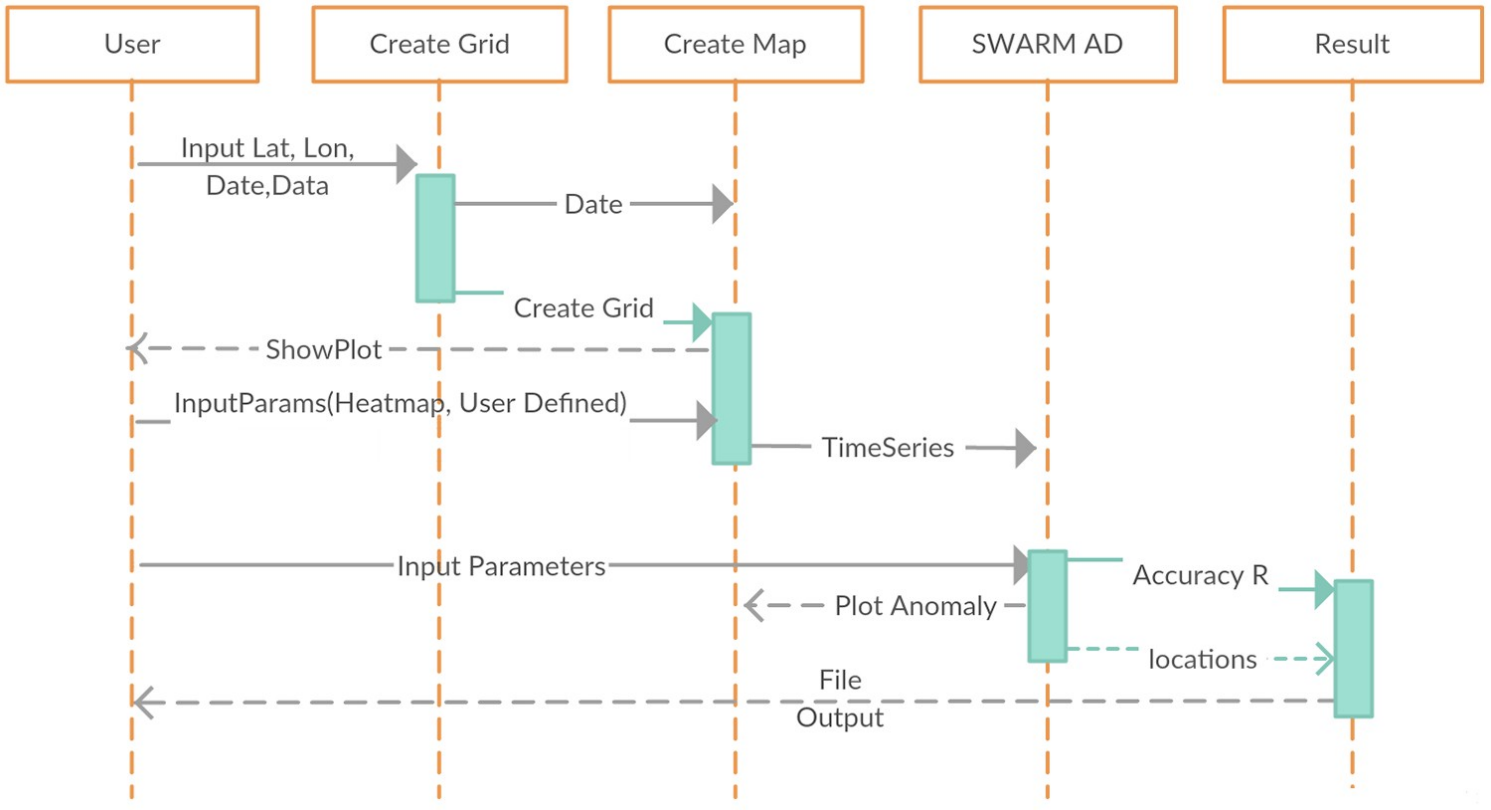
Sequence diagram of the proposed prototype.

A sequence diagram of the prototype is shown in Fig 6. The first step is the data extraction that is performed by the *Create Grid* function from the cdf files. This accepts four parameters, the longitude, latitude, radius and date of the seismic event. The date is required for the time series plots because it shows where the seismic event occurred in time. The plots are the different pre-processing methods implemented to eliminate the possibility of artificial anomalies.

Two of the pre-processing methods, the heatmap and the user defined mean require the user’s input before they return a result. If the selection is left empty then the default value of five data points is used to aggregate the first five data points in each date with measurements for both cases. The next stage is the AD stage. All of the AD methods make use of the last representational method used for pre-processing and perform AD in this particular time series sequence. The AD methods return their results to the plots printed by the pre-processing methods. As a final step an output file is also created. The output file shows the accuracy, the identified anomalous locations and the respective dates of the anomalies.

## 4 Experimental method

**Fig 7:**
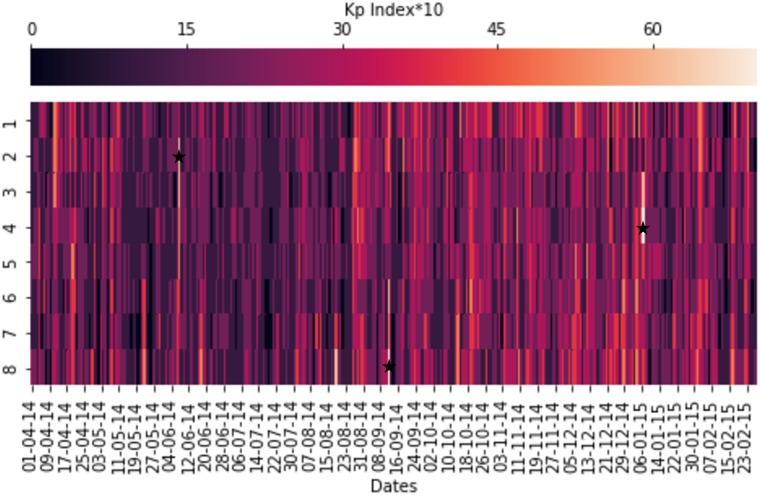
Planetary Kp-index, with the periods of high intensity annotated.

As a case study, we selected the Ludian earthquake with a scale of 6.2 M_w_. This earthquake occurred within the south-north seismic belt, mainland China, which was regarded as a strike-slip event, with the strike along 70° and 160° for the two nodal planes [36]. For the desirable evaluation criteria we need to make sure that we attribute the results to the right cause. For that reason we have to use two more regions in addition to the investigation. Any anomalies detected are expected to appear in this region. The second region is adjacent to the region of the seismic event and is regarded as the control region. This region is selected to eliminate the possibility of other seismic events causing EM anomalies because of “leakage” onto the other region. A third region has also been defined, which will be the ground truth and is a physically dormant region. The defined regions are the main control and ground truth (U.K.) and provide the experimental data. Their coordinates are shown in Table 2. Moreover, as part of our analysis, a useful indicator of classifying the anomalies is the Kp-index [37]. Kp index is estimated every 3 hours by measurements of the intensity of the magnetic field. In Fig 7, we have annotated the times of high intensity with a star symbol. They are the following: (a) 05-05-2014 00:00-03:00, (b) 22-06-2014 21:00-00:00 and (b) 19-08-2014 09:00-12:00.

**Table 2:** Investigated seismic regions and their coordinates.

Location, Magnitude, Depth (M_w_), (km)	Epicenter (Lat, Lon)	Control Region (Lat, Lon)	Ground Truth (U.K.) (Lat, Lon)
China, 6.2, 12	27.19° N 103.41° E	27.19° N 113.4° E	54.59° N 5.93° E

Based on the trajectory of the satellites the overpass of each from the predefined square grid can give a different number of readings. The number ranges from 5 to 64 per day or night cycle. Data extraction is not a simple task, the data has to be evaluated for the processing. The first issue identified with the data was the size of each square grid. We need as many data points as possible from each square grid to create a reliable and consistent time series sequence. However, the larger the square the higher the probability of interference from other sources. Increasing the resolution of the square is disproportionate with data quality. The optimum distance between the seismic event and the satellite projection was estimated using the Dobrovolsky formula $R=10^{0.43*M_{w}}$ [9] with M_w_ the magnitude scale, giving a proposed formula to select the Effective Distance in kilometers. Effectively, this was translated to cover ±5° in longitude and ±5° in latitude from the epicenter, making the grid 1000x1000km.

When we were confident about the validity of the measurements, the next step was to create the time series sequence. It is known that the satellites have a revisit period of 4 days on average, this leaves us with a gap of 4 days per each square, something that was not always the case. This also poses another significant problem. The satellite might not make a full pass over the grid every time. This creates inconsistent results as in each square we have a variable number of measurements. If a satellite does an incomplete pass through a square grid it provides less data points to work with. In order to make sure that no artificial anomalies are introduced, four aggregation methods are implemented, described in Section 3. In addition, with the removal of the main magnetic field with the CHAOS model, these variations are kept at a minimum.

The MFI has a daily oscillation called diurnal variation with a periodicity of almost a day [7]. The satellites pass in different times from each square and sometimes might pass twice during a given date. This creates another inconsistency problem because we have both day and night measurements within a square but the same does not occur in all square grids. Following that, we have to divide each date into day and night cycles. This process also creates more data points as described in the algorithm.

One last problem is concerned with missing data due to different reasons with the satellite instruments. This leads to null measurements, irregular passes, erroneous measurements etc. As a consequence the gap between two consecutive data points can be greater than 4 days. This can pose a problem for certain AD algorithms that work with periodic signals. This problem was addressed with the data pre-processing methods in stage 2 of the algorithm.

### 4.1 Experimental results and discussion

The choice of the algorithms to use is up for debate and there are many off the shelf algorithms. Two, the CE and the Fuzzy Shape-based method, were developed by ourselves and two are an implementation of already existing algorithms. They all have been extensively evaluated on benchmark and real datasets. The choice of their parameters is a direct result of the benchmark experiments. Data gathered by all three Swarm satellites were evaluated against four different AD methods in the selected study area.

Table 3 shows the parameters used for the algorithms. The parameters selected for all algorithms are based on extensive experimental research on real and benchmark data [22] and [20]. Both SAX and the Fuzzy Shape-based have a similar function range, hence they have the same parameter tuning. According to [21], the best 1D-SAX parameters are for slope 4 and PAA 2. The word length *w*, was kept in-line to the previous symbolic representational methods at 4. In algorithms the alphabet *a*, is hard-coded to the value of 3 based on the best experimental results suggested in [20].

**Table 3:** Parameters used for the AD methods.

	CE (K,λ)	Fuzzy (P,w)	1D-SAX (p,s,w)	SAX (p,w)
Parameters	(1,6)	(6,4)	(4,2,4)	(6,4)

**Fig 8:**
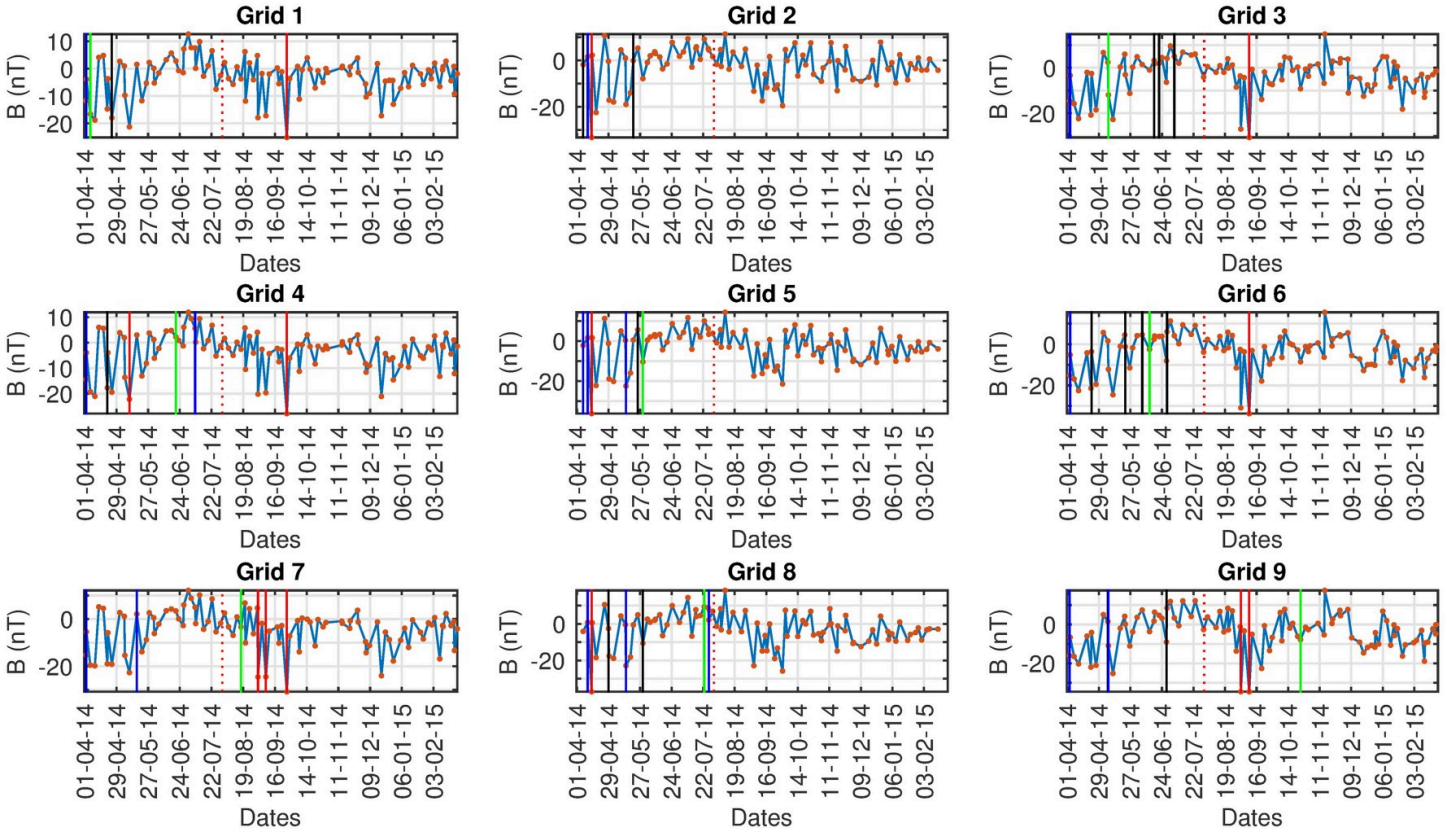
AD results Swarm A, *red: CE*, black: Fuzzy, *blue: D-SAX*, green: SAX.

**Fig 9:**
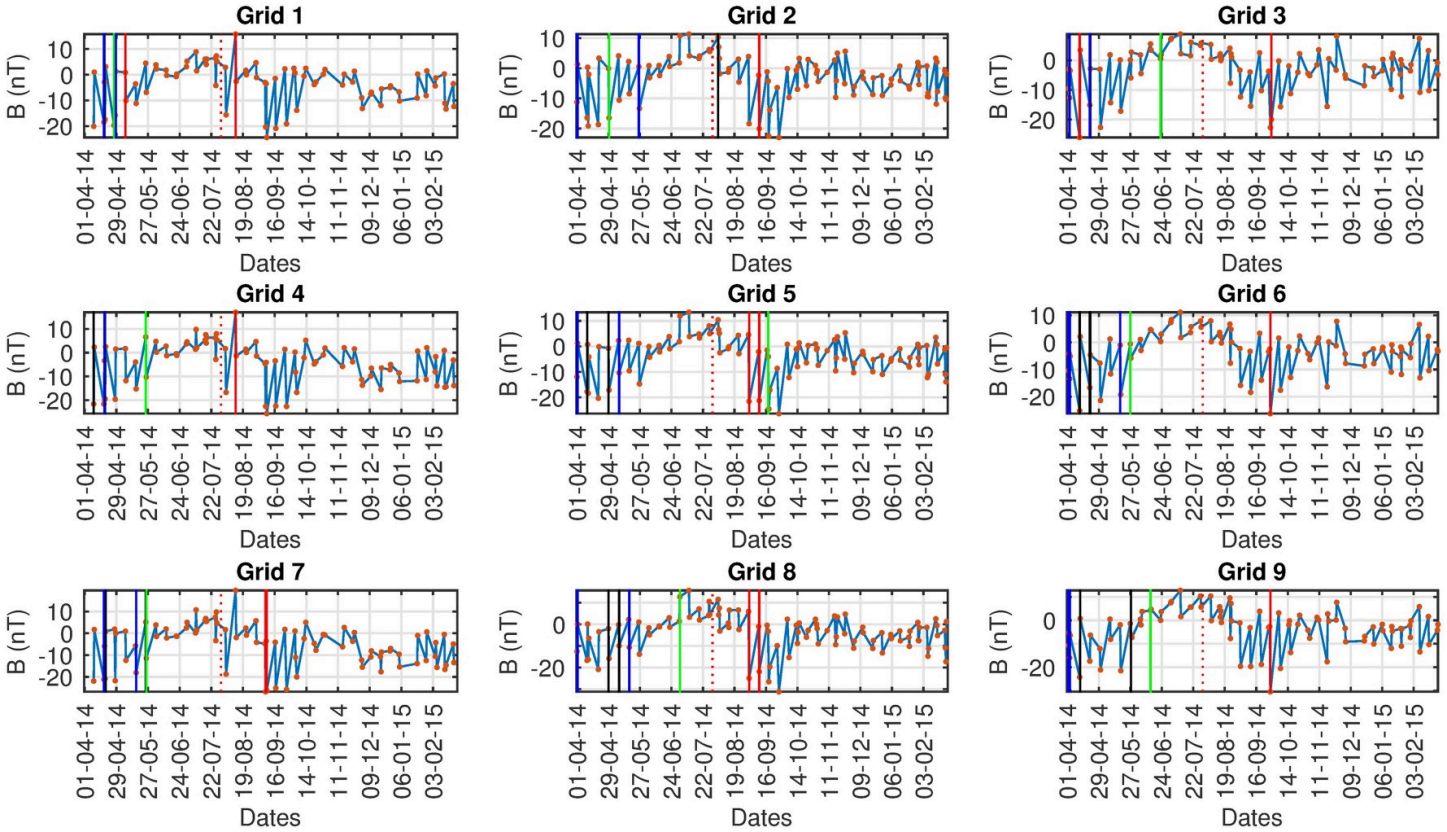
AD results Swarm B, *red: CE*, black: Fuzzy, *blue: D-SAX*, green: SAX.

**Fig 10:**
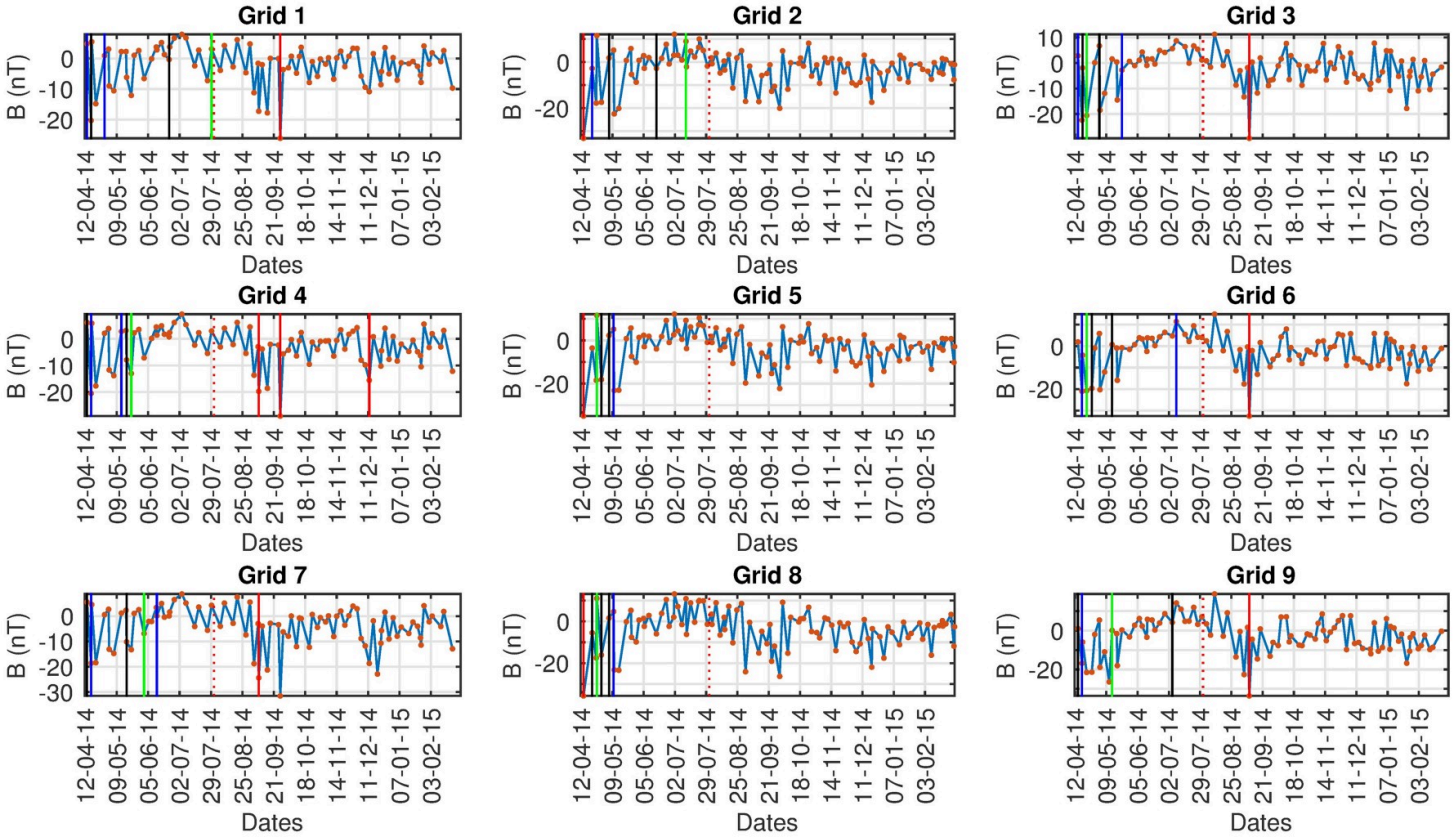
AD results Swarm C, *red: CE*, black: Fuzzy, *blue: D-SAX*, green: SAX.

Figs 8–10 show a common pattern among all algorithms used. All algorithms detect anomalies prior to the seismic event but we cannot confidently assess the impact because there are similar findings in both the control and ground truth regions. All algorithms detect anomalies even when we do not expect them, indicating that there is either: (i) a problem with the data collection, (ii) interference from other sources or causes (e.g solar activity) (iii) False Positives from the algorithms, (iv) other fore-shocks that were not taken into account originally or (v) a different region of interest has to be set with respect to boundaries/grids. Because of the aforementioned issues, nothing can be suggested about the results with certainty.

In Tables 4–6 the detected anomalous dates for the Ludian seismic event on 03/08/2014 by all algorithms are shown for Swarm A, B, C respectively. As seen a strong case against the data quality can be advocated. We know by empirical results that all the algorithms provide reliable and accurate results. Data sparsity poses a big problem and as we can see even in the main, control and ground truth region the algorithms have detected anomalies.

**Table 4:** Swarm A Grid 5, Ludian experimental results.

	Seismic	Control	Ground Truth
HOT-SAX	29-05-2014 (0%)	(0%)	(0%)
Fuzzy	17-05-2014 22-06-2014 30-07-2014 (0%)	10-6-014 08-07-2014 09-09-2014 (0%)	01-09-2014 10-09-2014 12-07-2014 (0%)
1D-SAX	12-12-2014 (0%)	10-10-2014 09-09-2014 (0%)	02-07-2014 (0%)
CE	14-04-2014 (0%)	10-05-2014 24-08-2014 (0%)	10-10-2014 08-08-2014 (0%)

**Table 5:** Swarm B Grid 5, Ludian experimental results.

	Seismic	Control	Ground Truth
HOT-SAX	18-09-2014 (0%)	12-12-2014 0(%)	26-07-2014 (0%)
Fuzzy	01-04-2014 21-04-2014 31-05-2014 03-07-2014 (0%)	10-10-2014 08-08-2014 (13%)	09-06-2014 13-10-2014 02-01-2015 (0%)
1D-SAX	01-04-2014 08-05-2014 (0%)	10-10-2014 08-08-2014 (0%)	10-09-2014 10-07-2014 04-06-2014 (0%)
CE	12-12-2014 10-09-2014 (25%)	10-08-2014 08-09-2014 (33%)	(0%)

**Table 6:** Swarm C Grid 5 Ludian, experimental results.

	Seismic	Control	Ground Truth
HOT-SAX	25-04-2014 (0%)	07-05-2014 12-12-2014 30-07-2014 (20%)	10-08-2014 (43%)
Fuzzy	18-05-2014 02-06-2014 (0%)	10-10-2014 25-08-2014 10-08-2014 (20%)	24-05-2014 04-11-2014 11-02-2015 (22%)
1D-SAX	14-Apr-2014 (0%)	05-04-2015 10-10-2014 13-07-2014 10-09-2014 (10%)	10-10-2014 08-08-2014 (40%)
CE	14-04-2014 15-05-2014 (0%)	10-10-2014 12-08-2014 (33%)	06-06-2014 01-02-2015 08-12-2014 (7%)

The R Metric, sometimes returns an accuracy, as seen in the brackets. Some detected anomalies fall within the algorithm’s predefined window. This means that an anomaly is detected before or after the date of the seismic event. However, it can not be used for evaluation purposes simply because the results are not consistent. The causes can not be directly attributed to a seismic event because the patterns are repeatable across all areas and satellites.

What is more, there is a visible trade-off in subdividing a region into smaller grids in order to better evaluate and locate anomalies, with that of a revisit period of 4 days. For a single satellite, there are not enough measurements for the precise monitoring of EM variations. Data interpolation techniques such as kriging [38] might provide a solution but with such sparse data it is difficult to use them in that stage. The creation of an accurate model of normality needs as much data as possible in order to provide high density and high resolution coverage. This will provide an almost continuous time series model that can be used to deliver consistent and confident results for our purpose. It should be noted that all these techniques introduce data loss and it is something that belongs to an entirely different field. In the end, these techniques are changing the landscape of the patterns of the original time series and must be used only when enough raw data is available.

Furthermore, the use of three identical satellites to provide measurements for a single region was originally thought to provide more data when combined. It would overcome the problem of monitoring the daily EM variation above a specific region. In contrast, because the satellites fly side-by-side this was not the case. Their trajectories instead of increasing data availability, produce data that were duplicates in terms of time and could only be used for validation purposes.

The problem is very constrained from data, meaning it needs data in very small time windows and time intervals to get a meaningful indication. In this respect, another problem is that one satellite’s measurements cannot be used to complement the others. Moreover, measurements of satellites A and C are of different intensity to B due to the satellites’ altitude and cannot be used to provide an immediate solution regarding data sparsity. Most of the issues are caused by the satellites’ data availability which is difficult to overcome. The amount of data available for each date also plays a significant role but it does not affect the processing as much as data availability. Nevertheless the results provide us with a clear picture of what needs to be done and what steps can be taken to overcome most of the problems. Overall, and that needs to be restated based on our findings, the main problem points to that of data quality. A unified data format, the combination of ground based sources with satellite, all within the same system can help solve the data problem and move forward to the critical stage of algorithm evaluation. The fact that we are not entirely confident about the data quality hampers all the subsequent stages of the processing, namely the algorithm evaluation.

## 5 Conclusion and future work

The work presented describes a system framework that provides a solution to: (i) extract data and select the region of investigation (ii) pre-processing with the aim of reducing artificial anomalies (iii) AD in Real world data and its evaluation with a new R metric. The system provides a pipeline for the automatic processing of Swarm data that was never explored before. It lays the foundation for an intuitive approach for the user to select a region and investigate EM variations as precursors to seismic events. It is a unique approach that bundles all three required functions together. The most important finding is that it reveals the problem faced with data gathered by the Swarm. Data sparsity not only in the Swarm constellation continues to be an insurmountable problem to establish a relationship between EM variations and seismic events. Further deep analysis is not possible and progress is slow despite some evidence for the opposite. Problems can also be traced back to the choice of algorithms. Nevertheless, it is important to keep in mind that these are two entirely separate issues.

Algorithm choice should be based both on non-periodic and periodic AD methods. The unknown nature of data and its irregular sampling have to be taken into account. In future work, more unsupervised algorithms such as Long-Short Term Memory (LSTM) [39] networks that have no parameter tuning can be used as a means to solve this problem. Furthermore, data collection continues to be a problem. A standard format for data products can advance the scientific understanding of anomalies and help streamline the data analysis. The combination of ground based and satellite data can be used in future studies and provide a more densely populated grid. Only by pinpointing the problem to data quality and addressing it can we confidently move to AD evaluation and that is what was proven in this study.

Potential problems can also be solved as in the case of Swarm by setting satellites into different orbits instead of side by side. The Zhangheng 1 (CSES-1) [40] satellite was launched in early February 2018 and will provide more data in that respect. Moreover, another satellite CASSIOPE [41] can also be used to increase the plurality of the data. Investigating and combining data from different satellites will provide a higher resolution in terms of data points and solve the main of the identified problems in this work. Addressing the data quality problem should be the first priority, with a careful pre-processing and data cleaning. In terms of detectable anomalies and their intensity, larger seismic events will be the focus of further research as the literature review suggests. Once the data cleaning problem is addressed, the focus should be on evaluating different data methods in an efficient and reliable manner.

## 6 Appendix

In this section functions and parameters used in our prototype are going to be defined.

**Grid**: The study area can be defined by the user’s input. In most research the study area is defined by using $R=10^{0.43*M_{w}}$ [9], that based on empirical observations. In our work, we create a square grid with dimensions 1000km x 1000km. This selection achieves enough resolution by enabling us to include as much data as possible without straying too far from the empirical observations. The central point of the grid is based on the latitude and longitude coordinates of a seismic event in China.

**PAA**: Piecewise Aggregate Approximation (PAA), is a dimensionality reduction method that works by simply aggregating data points. It is the core function of HOT-SAX and the Fuzzy Shaped-based method.

**SAX**: HOT-SAX [20] is a symbolic approximation and AD algorithm used as one of the AD schemes in our prototype. It originally has three input parameters, but in our implementation it uses two: (a) the PAA for the compression of the raw signal and (b) the word length, *w* of the subsequence to consider. Based on findings by the original authors an alphabet, *a*, of 3 yields the best results and it is hardcoded in our implementation.

**Fuzzy Shape-based method**: Introduced in [22], the Fuzzy Shape-based method uses a symbolic approximation based in three functions: (a) the shape, (b) the PAA based on equally segmented areas, and (c) the PAA that segments the hyperplane according to a Gaussian distribution based on equiprobable segments.

**CUSUM-EWMA**: A statistical method introduced in [23], is a product of two distinct algorithms, the CUSUM and the EWMA. Two parameters, the CUSUM statistic *K* and the number of the historical values the algorithm is going to consider, also known as EWMA statistic, λ are used.

From a statistical point of view, the CUSUM-EWMA (CE) is not constrained by the length of the anomaly. However, it also requires parameter tuning. The overall objective is to illustrate how different AD methods perform in a constrained real-world environment with sparse data. All algorithms are compared by using a novel metric, *R*, introduced in [42] which takes into account the subsequence length, the predicted anomalous location by the algorithm and the true anomalous location.

**1D-SAX**: An improvement upon HOT-SAX presented in [21], accepts the slope of each subsequence as an additional and more accurate symbolic representational parameter. The algorithm therefore uses three parameters: (a) the PAA, the slope, *s* and the word length *w* for each subsequence.

**R Metric**: All algorithms are evaluated using a novel metric, *R* with Real world data using the metric defined in [42]. It is a unique metric because it uses a pre-defined length to measure the accuracy of each AD method and is specifically tuned to account for the localization of the anomaly. A predefined length is what research has shown to be an appropriate time to expect precursory signals before the occurrence of a seismic event. In other words it measures the accuracy between the expected actual anomaly (date/time of the seismic event) and the identified anomaly by the algorithm.
